# Preliminary assessment of *Hedychium coronarium* essential oil on fibrinogenolytic and coagulant activity induced by *Bothrops* and *Lachesis* snake venoms

**DOI:** 10.1186/1678-9199-20-39

**Published:** 2014-09-01

**Authors:** Cíntia A SF Miranda, Maria G Cardoso, Mariana E Mansanares, Marcos S Gomes, Silvana Marcussi

**Affiliations:** 1 Department of Chemistry, Federal University of Lavras (UFLA), Lavras, Minas Gerais State, Brazil; 2 Department of Biology, Federal University of Lavras (UFLA), Lavras, Minas Gerais State, Brazil; 3 Laboratório de Química Orgânica, Departamento de Química, Universidade Federal de Lavras, Caixa postal 3037, CEP 37200-000 Lavras, MG, Brasil

**Keywords:** Volatile oils, Weeds, Natural inhibitors, Antivenom therapy

## Abstract

**Background:**

The search for new inhibitors of snake venom toxins is essential to complement or even replace traditional antivenom therapy, especially in relation to compounds that neutralize the local effects of envenomations. Besides their possible use as alternative to traditional antivenom therapy, some plant species possess bioactive secondary metabolites including essential oils, which can be extracted from weeds that are considered substantial problems for agriculture, such as *Hedychium coronarium*.

**Methods:**

The essential oils of leaves and rhizomes from *H. coronarium* were extracted by hydrodistillation, and their potential inhibitory effects on the coagulant and fibrinogenolytic activities induced by the venoms of *Lachesis muta*, *Bothrops atrox* and *Bothrops moojeni* were analyzed. Citrated human plasma was used to evaluate the clotting time whereas changes in fibrinogen molecules were visualized by electrophoresis in polyacrylamide gel. The experimental design used for testing coagulation inhibition was randomized in a 3 × 2 factorial arrangement (concentration × essential oils), with three replications. The essential oils were compared since they were extracted from different organs of the same botanical species, *H. coronarium*.

**Results:**

The results suggest that the oils interact with venom proteases and plasma constituents, since all oils evaluated, when previously incubated with venoms, were able to inhibit the clotting effect, with less inhibition when oils and plasma were preincubated prior to the addition of venoms.

**Conclusions:**

Thus, after extensive characterization of their pharmacological and toxicological effects, the essential oils can be used as an alternative to complement serum therapy, especially considering that these plant metabolites generally do not require specific formulations and may be used topically immediately after extraction.

## Background

Weeds are a serious threat to the conservation of native plants and cultivated crops because they alter the balance of ecosystems, with consequent damage to agriculture. These plants have been removed by physical methods, including burning and hoeing, or more commonly by chemical methods such as the use of synthetic herbicides [1]. Although efficient from a functional point of view, these agrochemicals present low selectivity, acting indiscriminately on invasive and cultivated species while changing them morphologically and physiologically. These alterations hamper the determination of current and especially future changes to the animal species that consume these plants [2].

Control of weeds by manual hoeing at ideal times does not reduce crop yields in relation to the use of herbicides [1]. Considering this, it is important to propose an alternative destination for weeds, one that is economically and ecologically sustainable, such as the exploitation of the biological potential of their secondary metabolites, among which we highlight the essential oils.

Essential oils are complex mixtures of volatile, lipophilic and odoriferous substances from various plant tissues. They are composed primarily of terpenoids, predominantly monoterpenes, sesquiterpenes and their oxygenated derivatives such as alcohols, ketones, aldehydes, esters, phenols and oxides. These metabolites play essential biological functions for survival and adaptation of the plant to the environment, as well as present pharmacological activities of medical-scientific interest [3].

Standing out among the invasive exotic species is *Hedychium coronarium*, an aquatic monocot belonging to the botanical family Zingiberaceae, widely distributed in Brazil and adapted to cerrado fields, coastal forests of Serra do Mar, araucaria rainforests, coastal and inland forests of Bahia, caatinga and Upper Paraná Atlantic forests [4].

The biological potential of essential oils extracted from rhizomes of this species has been widely studied including reports of their antibacterial, antifungal, larvicidal and antioxidant activities [5-9]. To the best of our knowledge, research with essential oils extracted from leaves is restricted to a single work, which reports antioxidant, larvicidal, antifungal and antibacterial activities [9]. In addition, this is the first study that evaluates the properties of oils extracted from the rhizomes and leaves of the species *H. coronarium*, by analyzing their potential pharmacological effects on coagulation and fibrinogenolysis related to snake envenomation. The results described herein represent a screening of the inhibiting activity of such oils against effects induced by snake venom proteases. This partial characterization may be used to guide further studies, especially *in vivo*, aiming at future applications of these oils.

Snake envenomations comprise a public health issue in Brazil that deserves the attention of health authorities, and mainly affects rural workers [10]. Snakes of the *Bothrops* genus are responsible for most accidents in Latin America, although the *Lachesis* genus, widely distributed in humid regions such as the remote rainforest areas in Central America and South America, is responsible for accidents of equal severity, resulting in prominent local effects, permanent sequelae and even death [11].

Snake venoms are composed mainly of proteins with enzymatic activity that belong to different classes, such as L-amino acid oxidases, phospholipases A_2_, serine proteases and metalloproteases, the two latter being primarily responsible for effects on hemostasis [11]. The combination of these protein compounds is directly related to the damage caused by these venoms, whose pathophysiology includes local effects (intense pain, edema, hemorrhage and necrosis) and/or systemic effects, such as nausea, coagulopathy, hypotension, cardiovascular shock and kidney disorders [12].

Although their use for the treatment of snakebites is traditional in many countries, plant extracts have been shown empirically to be a promising alternative for this purpose, but without scientific evidence of their efficacy [13]. Several studies are speculating on the use of plant extracts as sources of molecular prototypes which can be used in the treatment of snake envenomations, or to complement the traditional antivenoms, which have shown little effectiveness in minimizing the local damage [3,13-19]. However, studies employing essential oils for that purpose are still scarce [18].

With that in mind, the present study aimed to evaluate the inhibitory potential of essential oils extracted from leaves and rhizomes of *H. coronarium* species against the coagulant and fibrinogenolytic activities induced by *Lachesis muta*, *Bothrops atrox* and *Bothrops moojeni* snake venoms.

## Methods

### Collection and registration of plant material

Specimens of the plant species *Hedychium coronarium* were collected at the Federal University of Lavras (UFLA), in Lavras, MG (21° 14 ′S, longitude 45° 00′ W Gr and 918 m altitude), at around 8:00 am on February 25^th^, 2012. Young leaves (rib and limb) and rhizomes of mature plants at the stage of full flowering were harvested. Species identification was kindly performed by Dr. Mariana Esteves Mansanares, Department of Biology of UFLA and exsiccates were deposited in the ESAL Herbarium at UFLA under the registration number 26942.

### Essential oil extraction

The essential oils from fresh leaves were extracted by steam distillation using a modified Clevenger apparatus, adapted to a round-bottom flask with a capacity of 4 liters, for a period of two hours [20]. Resulting samples were centrifuged for five minutes at 965.36 *g*, and the essential oils were separated with a Pasteur pipette, disposed in amber glass bottles and stored at 4°C. The composition of the essential oils was determined by Miranda [21]. The oil volumes used during the analyses were selected from the results obtained in toxicity tests (results not shown).

### Obtaining human citrated plasma

Blood samples were obtained from the researchers involved in the work, who presented good health and normal exams of bleeding time (BT), clotting time (CT), prothrombin activity time (PAT), and partially activated thromboplastin time (PATT). The samples were collected in BD Vacutainer® (Becton, Dickinson and Company, USA) tubes with buffered sodium citrate 0.109 mol and 0.105 mol (3.2%) in the proportion of nine parts of blood to one part of citrate solution, as recommended by the Clinical and Laboratory Standards Institute (CLSI). The original project whose present work is included was approved by the Ethics Committee for Human Research of UFLA, protocol number 09978312.8.0000.5148.

### Snake venoms

Crystallized venoms corresponding to a single specimen of each snake species (*Lachesis muta*, *Bothrops atrox* and *Bothrops moojeni*) were purchased from Bio-Agents (Serpentário Proteínas Bioativas Ltda., Batatais, Brazil), and stored at 4°C until used in the biological assays. At this point, venoms were dissolved in phosphate buffered saline (PBS) at different concentrations (10, 5, 1 and 0.5 mg/mL).

### Effects of essential oils on coagulation: induction and/or inhibition of clot formation

The coagulant capacity of essential oils was previously evaluated with adaptations related to the volumes of samples evaluated [22]. Volumes of 200 μL of citrated human plasma had their temperatures previously stabilized in a water bath at 37°C. Then, essential oils were added separately in tubes at different volumes (0.6 and 1.2 μL) and the reaction mixtures remained under observation at 37°C for 24 hours. The observation was continued by gentle agitation every five minutes for a period of 45 minutes, and if no coagulation of the plasma was detectable, this observation was extended for a period of 24 hours, to define plasma uncoagulability.

The inhibitory action of oils on the coagulant activity induced by snake venoms was assessed with adaptations. Pilot tests were conducted to determine the concentration of each venom able to coagulate 200 μL of citrated plasma in a time interval between 40 and 120 seconds at 37°C.

We evaluated two possible mechanisms of interaction for the oil molecules: firstly, with fibrinogen, and, secondly, with snake venom proteases. Accordingly, the first assay was carried out by preincubating plasma (200 μL) with essential oils (0.6 and 1.2 μL) at 37°C for 15 minutes, followed by the addition of 1 μL (10 μg) of each venom (*L. muta*, *B. atrox* and *B. moojeni*) separately, measuring the time for the formation of a rigid clot. The second assay was performed by preincubation of essential oils (0.6 and 1.2 μL) with the same venoms (10 μg; 50 μg/mL) for 15 minutes at 37°C, followed by the addition of plasma (200 μL) and measurement of the clotting time.

### Effects of essential oils on the fibrinogen structure and the fibrinogenolytic activity induced by venoms

The activities were carried out with modifications [23]. The essential oils (0.6 and 1.2 μL) were incubated with bovine fibrinogen (60 μg) for 90 minutes at 37°C in a final volume of 25 μL (PBS). The reactions were stopped by addition of 10 μL of bromophenol blue solution [0.05 M Tris-HCl, pH 8.0, comprising 10% (v/v) glycerol, 10% (v/v) mercaptoethanol, 2% (w/v) sodium dodecyl sulfate (SDS) and 0.05% (w/v) bromophenol blue] and boiling the samples for five minutes in a water bath. The samples were analyzed by polyacrylamide gel electrophoresis at 12% (acrylamide: bisacrylamide, 19:1) in the presence of SDS (SDS-PAGE) under denaturing conditions [24]. A sample control containing only fibrinogen was used for the visualization of the band pattern corresponding to the α, β and γ chains of fibrinogen.

The inhibitory potential of the essential oils on the proteolytic action of *L. muta*, *B. atrox* and *B. moojeni* venoms was assessed by SDS-PAGE after preincubation of samples. We evaluated possible interactions between the essential oils (0.6 and 1.2 μL) and proteases present in the venoms (30 μg) by incubating them (final volume of 25 μL; PBS) for 30 minutes at 37°C with subsequent addition of fibrinogen (60 μg) and incubation for 90 minutes at the same temperature. Possible interactions between oil molecules and fibrinogen were considered in another assay, by preincubating bovine fibrinogen (60 μg) and essential oils (0.6 and 1.2 μL) for 30 minutes at 37°C with subsequent addition of each venom (*L. muta*, *B. atrox* and *B. moojeni*; 30 μg) and incubation for 90 minutes at the same temperature.

The reactions were stopped by addition of 10 μL of bromophenol blue solution and boiling for five minutes in a water bath, followed by analysis on SDS-PAGE under denaturing conditions [24].

### Statistical analysis

The experimental design used for testing the inhibitory action on coagulation was randomized in a 3 × 2 factorial arrangement (concentration × essential oils), with three replications. The essential oils were compared since they are extracted from different vegetal organs of the same botanical species, *H. coronarium*. Significant factors by the F test (p < 0.05) were tested for average (Scott-Knott 5%) for the determination of the models. Data were analyzed using the statistical analysis system variance for balanced data Sisvar described by Ferreira [25].

## Results and discussion

Miranda [21] identified 99.2 and 99.3% of the chemical constituents of the essential oils of rhizomes and leaves of *H. coronarium*, respectively, with the two oils being composed entirely of terpenoids. Among these terpene constituents, the oil extracted from rhizomes showed 98.2% monoterpenes and 1% sesquiterpenes, while that from leaves presented 84.3% monoterpenes and 14.9% sesquiterpenes. Among the major compounds of the oil from rhizomes are the monoterpenoids β-pinene (41.5%), 1.8-cineole (23.6%) and α-pinene (13.1%), whereas leaf oil presents the monoterpenoids β-pinene (46.9%) and α-pinene (19.2%), and the sesquiterpene β-caryophyllene (13.2%). Such variability observed in the chemical compositions of the essential oils of leaves and rhizomes of a botanical species is commonly observed, due to the fact that the location and distribution of secondary metabolites occur in a characteristic manner between different parts of the same plant [26]. Additionally, the individual chemical compositions of essential oils directly influence the biological activities and pharmacological effects of these secondary metabolites.

The search for natural products with antiophidian properties has motivated studies to characterize the toxic and pharmacological properties of extracts and essential oils from several plant species, attributing major economic and ethnopharmacological importance to the secondary metabolites. In this context, we highlight the widespread use of popular plant species with antiophidian properties, capable of neutralizing the local effects of venoms, the lack of studies that investigate the antiophidian potential of essential oils and the importance of exploring the pharmacological potential of secondary metabolites extracted from invasive species, making this an innovative and valuable scientific research area.

Thus, the study of the antiophidian properties of essential oils extracted from rhizomes and leaves of *H. coronarium*, such as their inhibitory effects on coagulation and fibrinogenolysis induced by the venoms of *L. muta*, *B. atrox* and *B. moojeni*, evaluated in the present work, may result in information that indicates the use of these compounds not only in the treatment of snakebites but also in the therapy of diseases responsible for hemostatic disorders or inflammatory processes. In addition, new studies that will complement the functional characterization of oils are being conducted based on the evaluated experimental conditions and the results observed in this study.

The essential oils showed no effectiveness in inhibiting the fibrinogenolysis induced by the venoms evaluated, when previously incubated with venoms or fibrinogen.

Throughout the 24-hour observation period, the essential oils tested did not cause coagulation of citrated human plasma at the volumes tested (0.6 and 1.2 μL), thus indicating a lack of coagulant potential.

**Figure 1:**
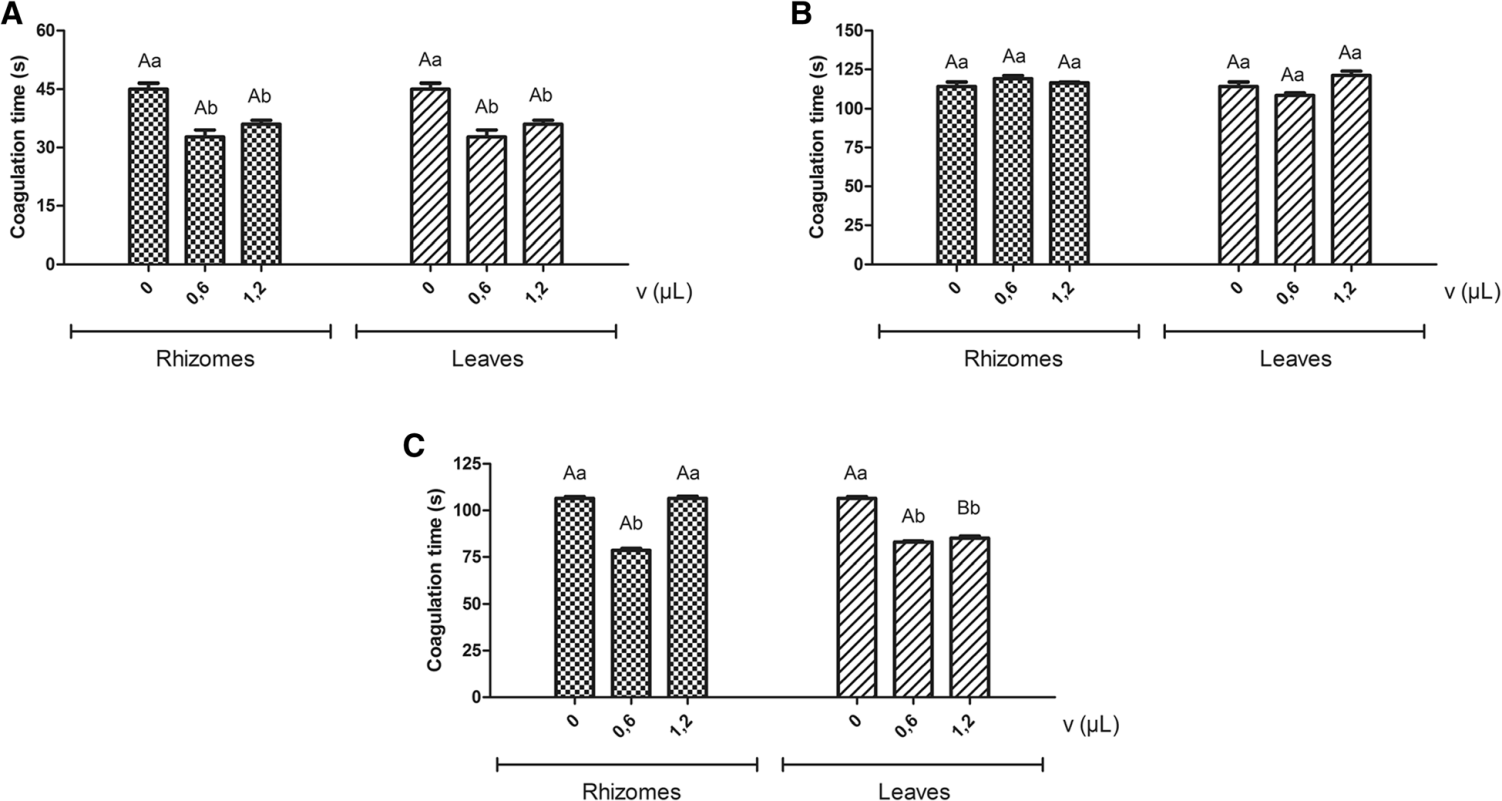
**Effect of essential oils on the coagulation of human plasma induced by *Bothrops *and *Lachesis *snake venoms after previous incubation of citrated human plasma at 37°C with different volumes of essential oils extracted from rhizomes and leaves of *H. coronarium*, followed by addition of the venoms of *Lachesis muta*, *B. moojeni *and *B. atrox *(50 μg/mL). (A) ***Lachesis muta* inducing clotting. **(B) ***Bothrops moojeni* inducing clotting. **(C) ***Bothrops atrox* inducing clotting. Mean values of the clotting time followed by the same letter, in uppercase to compare the essential oils analyzed and in lowercase for comparison between volumes of the same essential oil, do not differ significantly at 5% probability by the Scott-Knott test.

By previously incubating citrated human plasma at 37°C in the presence of essential oils from rhizomes and leaves of *H. coronarium*, with subsequent addition of the venoms of *L. muta* and *B. atrox*, a decrease in the clotting times induced by the venoms could be observed. For *L. muta* venom (CT = 45.0 ± 1.5 seconds), clotting times of 32.7 ± 1.8 and 36.0 ± 1.0; 35.7 ± 0.7 and 40.5 ± 0.8 (corresponding to oil volumes of 0.6 and 1.2 μL) for oils of rhizomes and leaves, respectively, were observed. For *B. atrox* venom (CT = 106.5 ± 0.9 s), the oil from leaves presented significant changes, with clotting times of 83.1 ± 0.6 and 85.2 ± 1.1, corresponding to oil volumes of 0.6 and 1.2 μL, and 78.6 ± 1.0 for the rhizome oil volume of 0.6 μL. On the other hand, the oils from the different parts of *H. coronarium* did not statistically alter the clotting time induced by the venom of *B. moojeni* (Figure 1).The data presented in Figure 1 show a procoagulant action previously unreported in the literature with regard to the effects of antiophidian plant extracts. This is possibly due to the structural variability of molecules, mainly of hydrophobic character, present in essential oils and absent in most aqueous or hydroalcoholic extracts described in the literature. The results suggest that the oils possibly interact with plasma proteins involved in the coagulation cascade, making them more susceptible to the proteolytic action of venoms and consequently accelerating the plasma coagulation. This interaction probably occurs between individual constituents of the essential oils and proteins such as thrombin, fibrinogen or fibrin, which are the major targets of coagulant toxins, thereby acting as procoagulants.

Given the limitations of these tests, such as the maximum sample volume in relation to the plasma volume and limited available quantities of oils for the assays, future studies should be performed using variations in incubation times, volumes of plasma, oils and venoms, as well as the analysis of the effects of oils on isolated toxins.

**Figure 2:**
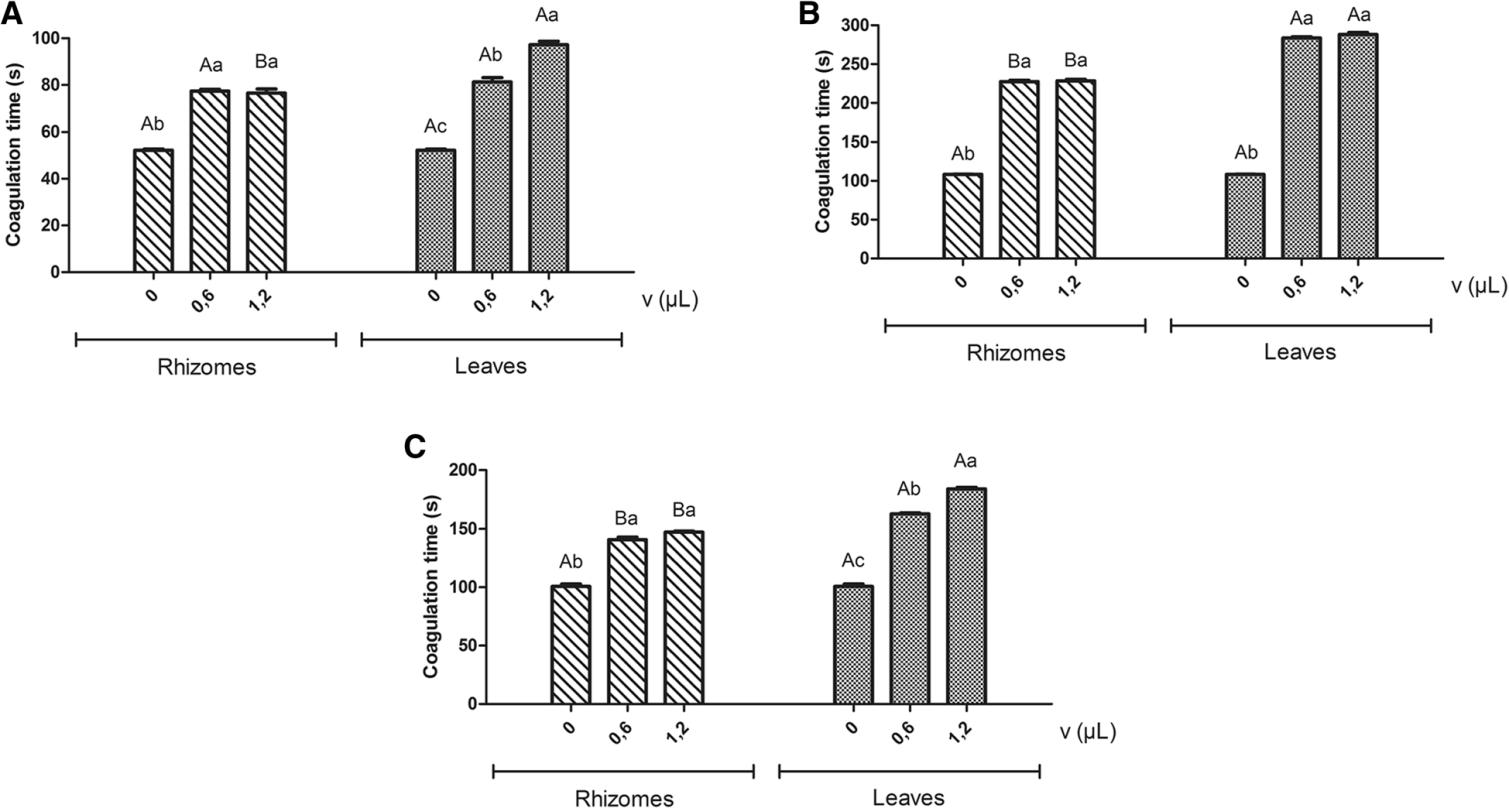
**Effect of essential oils on the coagulation of the human plasma induced by *Bothrops *and *Lachesis *snake venoms after previous incubation of *Lachesis muta, B. moojeni *and *B. atrox *(50** μ**g/mL) snake venoms at 37°C with different volumes of essential oils extracted from rhizomes and leaves of *H. coronarium*, followed by addition of citrated human plasma. (A) ***Lachesis muta* inducing clotting. **(B) ***Bothrops moojeni* inducing clotting. **(C) ***Bothrops atrox* inducing clotting. Means followed by the same letter, in uppercase for comparison between the essential oils analyzed and in lowercase to compare volumes of the same essential oil, do not differ significantly at 5% probability by the Scott-Knott test.

By analyzing the effect of preincubation of snake venoms with the essential oils on the clotting time of citrated plasma, we observed that the oils from rhizomes and leaves of *H. coronarium* were able to extend the clotting time induced by all the venoms evaluated, being potential anticoagulants. Additionally, the results showed that the essential oil from leaves extended plasma coagulation time induced by all three venoms evaluated at higher levels than those obtained from rhizome oil (Figure 2).

The clotting time of *L. muta* venom (52.2 ± 0.5 seconds) was extended by the oil from leaves to 81.3 ± 1.8 and 97.2 ± 1.5, corresponding to volumes of 0.6 and 1.2 μL, respectively. The clotting time of *B. atrox* (100.8 ± 2.0) was extended by the same oil to 162.9 ± 0.8 and 183.9 ± 1.3 μL, respectively.

Statistically significant variations in clotting times were produced by employing two different volumes (0.6 and 1.2 μL) of the essential oil of leaves, with no significant differences between the results obtained with the same volumes of rhizome oil. The oil obtained from rhizomes, when previously incubated with venoms, prolonged clotting times induced by *L. muta* to 77.4 ± 07 and 76.5 ± 1.8, and by *B. atrox* to 140.7 ± 2.2 and 147.3 ± 0.7, with values corresponding to volumes of 0.6 and 1.2 μL, respectively. Preincubation of oils from rhizomes and leaves with venoms before addition of the plasma resulted in greater inhibition of the *B. moojeni* venom-induced clotting effect in comparison to the tests with preincubation of the oils with the plasma. The clotting time induced by *B. moojeni* venom (108.3 ± 0.4) was prolonged to 227.4 ± 1.6 and 283.5 ± 1.5 for the oil volume of 0.6 μL, and to 228.3 ± 2.1 and 288.0 ± 2.5 for 1.2 μL, corresponding to oils from rhizomes and leaves, respectively (Figure 2).

These findings suggest possible interactions between terpene compounds present in the oils and proteases present in the venoms, which are responsible for their clotting effect. Considering the different clotting times obtained with the different oils and different snake venoms, an inhibition pattern could not be detected, so that several terpenes may have been responsible for specific interactions with different toxins present in the venoms evaluated. Possible inhibition mechanisms may include the scavenging of ion cofactors by the oils, terpenes binding to specific catalytic sites or acting as ligands of enzymatic cofactors, and terpenes interacting with hydrophobic amino acids of the toxins, changing their three dimensional conformation, solubility and hence interfering with their proteolytic activity.

Future trials to evaluate the effects of oils and their major isolated constituents on molecules or classes of venom toxins can provide accurate information about the interactions, predicting specificity and inhibition mechanisms. The biological effects of essential oils are rarely associated with a single constituent, but rather with a group of secondary metabolites, which corroborates the observations made in this work [27]. This differentiated interaction of essential oils from leaves and rhizomes of *H. coronarium* with the venoms of *L. muta*, *B. moojeni* and *B. atrox*, in which the oil from leaves induced higher inhibition of coagulation, suggests that its higher content of sesquiterpenoids (14.9% in comparison to merely 1% in the oil from rhizomes) may account for greater antioxidant efficiency and may be associated with the inhibition of toxins that exert pathophysiological effects partially attributable to oxidative actions.

In general, the composition of venoms present minor variations among species of the same genus (e.g. *B. atrox* and *B. moojeni*) and higher among species of different genera (e.g. *B. atrox* and *L. muta*). Nevertheless, the results contradict this observation, suggesting the presence of homologous proteases in the venoms of *B. atrox* and *L. muta*, largely responsible for the induction of coagulation and absent in the venom of *B. moojeni*. Differences in the chemical compositions of venoms are related not only to distinct genera and species, but also to the age, sex and geographic region of the animal, this latter being related to differences in diet, climate, and other factors [28].

The effect of venoms on blood hemostasis is mainly associated with the action of proteases, which can act on factors of the beginning of the coagulation cascade, as well as on fibrinogen, thrombin, fibrin and on platelet surface receptors [29-36]. These various targets for proteases hamper the development of hypotheses on the action mechanisms of natural inhibitors, such as the essential oils evaluated in the present study.

There are few reports in the literature on the antiophidian properties of essential oils. The inhibitory effect of extracts and essential oils from *Nectandra angustifolia* leaves on the hemolysis and coagulation induced by *B. neuwiedi* venom were evaluated, showing that the essential oil was only effective in inhibiting coagulation [18]. The monoterpenes α-pinene, β-pinene and limonene were the major constituents identified in the essential oils evaluated and were considered by these authors as possible coagulation inhibitors. Additionally, these authors expressed the need for further analysis of the active components of these oils, since there is no conclusive data on mechanisms of specific interactions between plant and venom molecules.

Fibrinogen, a dimeric plasma glycoprotein of 340 kDa composed of six polypeptide chains (α, β, γ), participates in the coagulation cascade, and is converted by thrombin to fibrin monomers [37]. This protein has been extensively used for the evaluation of the potential fibrinogenolytic effects of snake venoms, and for the verification of potential antiophidian properties of natural compounds, mainly of plant origin [3,13,15-18,38,39].

The essential oils from rhizomes and leaves of *H. coronarium* showed no proteolytic effect on fibrinogen. These essential oils were also unable to inhibit the fibrinogenolysis induced by the venoms of *L. muta*, *B. moojeni* and *B. atrox*, since electrophoretic migration profiles observed for samples of fibrinogen incubated with venoms were identical to those obtained with venoms previously submitted to incubations in the presence of oils (results not shown). These evaluations were performed with preincubation of oils (0.6 and 1.2 μL) with fibrinogen and subsequent addition of venoms, and/or with previous incubation of oils and venoms and subsequent addition of fibrinogen.

These results suggest that the compounds present in the oils may interact with specific non-fibrinogenolytic proteases involved in the coagulation disorders promoted by snake envenomations, thus ruling out a number of metalloproteases described in the literature, especially those isolated from the venoms of *B. atrox* (batroxase, BaTX-I botroxostatine and atroxlysin-I) and *B. moojeni* (BMMP-III and BthMP) [30,31,33,34,40,41] and highlighting the serineproteases mainly responsible for such coagulation disorders [35,36,41-45].

The potential neutralization of different parts (leaves, stems and roots) of fresh and dried *Mikania glomerata* aqueous extracts on the enzymatic and pharmacological effects of *Bothrops* and *Crotalus* snake venoms were evaluated [13]. All extracts were active in inhibiting the hemolytic, fibrinogenolytic and coagulant activities of the venoms; however, the intensity of the inhibitions showed specificity in relation to each extract acting on each activity evaluated. The individuality observed in these activities in the present work, may be due to the fact that the metabolites are distributed in a particular manner in each organ of the same plant to constitute a characteristic chemical composition of each extract or essential oil that is crucial for its pharmacological activity [26].

## Conclusions

Although the anticoagulant action mechanisms of the essential oils evaluated are still unknown, they significantly inhibited coagulation induced by the snake venoms tested, which suggests that they could be used as an alternative to complement the available antivenoms, especially considering that essential oils do not require specific formulations and their topical use may be performed immediately after extraction. In addition, snake venoms have been widely used in studies of normal physiological mechanisms and of development of various diseases, considering that many human enzymes are structurally and functionally similar to molecules present in venoms, indicating the importance of the characterization of new natural compounds capable of interacting with different classes of snake toxins (metalloproteases, serine proteases, phospholipases A_2_, L-amino acid oxidases, etc.), thus directing scientific basis for the development of new therapies. Therefore, this work is an investigative and bioprospecting basic research and further *in vivo* assays should be performed. We believe that natural products may be used to treat or combat snake envenomations and/or in the therapy of diseases responsible for inflammatory and homeostatic processes disorders.

### Ethics committee approval

The present study was approved by the Ethics Committee for Human Research of UFLA, protocol number 09978312.8.0000.5148.

## Competing interests

The authors declare that there are no competing interests.

## Authors’ contributions

CASFM and SM designed and performed the experiments and wrote the manuscript. MSG participated in the design of the study and performed the statistical analysis. MGC discussed the experimental design and results with the other authors and participated in manuscript writing. MEM performed the species identification. All authors read and approve the final manuscript.
